# Effects of Etching Variations on Ge/Si Channel Formation and Device Performance

**DOI:** 10.1186/s11671-018-2631-1

**Published:** 2018-07-31

**Authors:** Jiann-Lin Chen, Yiin-Kuen Fuh, Chun-Lin Chu

**Affiliations:** 1 Department of Mechanical and Automation Engineering, I-Shou University, Kaohsiung City, 840 Taiwan; 2 Department of Mechanical Engineering, National Central University, Chung-li, 300 Taiwan; 3 National Nano Device Laboratories, Hsinchu, 300 Taiwan

**Keywords:** Ge gate-all-around, High-defect, Dry etching, Numerical simulation

## Abstract

During the formation of Ge fin structures on a silicon-on-insulator (SOI) substrate, we found that the dry etching process must be carefully controlled. Otherwise, it may lead to Ge over-etching or the formation of an undesirable Ge fin profile. If the etching process is not well controlled, the top Ge/SOI structure is etched away, and only the Si fin layer remains. In this case, the device exhibits abnormal characteristics. The etching process is emerging as a critical step in device scaling and packaging and affects attempts to increase the packing density and improve device performance. Therefore, it is suggested that optimization of operating the plasma reactor be performed through simulations, in order to not only adjust the process parameters used but also to modify the hardware employed. We are going to develop Ge junction-less devices by employing updated fabrication parameters. Besides, we want to eliminate misfit dislocations at the interface or to reduce threading dislocations by applying cyclic thermal annealing process to meet the goal of obtaining suspended structure of epitaxial Ge layers with high quality.

## Background

One way of increasing the response speed of semiconductor devices and reducing their power consumption is to use a semiconductor with a high-carrier mobility. Examples of such materials include Ge as well as its alloys and compounds. However, when a structure is formed by stacking a layer of a semiconductor material on top of a substrate of a different semiconductor, problems may arise. For example, the difference in the lattice sizes of the semiconductor substrate and the overlying layer of a different semiconductor material may cause dislocations, which may have an adverse effect on device performance. Conventionally, in order to fabricate Ge-based semiconductor devices that include a Si substrate, a thicker buffer layer or sacrificial layer (e.g., a Si/Ge buffer layer) is formed on the silicon substrate. Subsequently, a Ge epitaxial layer is grown on the Si/Ge buffer layer. Next, anisotropic and isotropic etching processes are performed sequentially to remove a part of the buffer layer and retain the Ge epitaxial layer. VLSI devices must show a high drive current, low off-state leakage current, and low supply voltage, in order to ensure high performance, including low standby power consumption and reduced dynamic power dissipation. Currently, strain-enhanced mobility, high-k/metal gate, and three-dimensional (3D) device architecture at the 22 nm node [1] are some of the technologies being used for the continuous scaling of complementary metal-oxide-semiconductor (CMOS) devices with a Si channel. In addition to characteristics such as high mobility, new device architectures such as those of gate-all-around (GAA) [2] and ultrathin-body field-effect transistors (FETs) [3] are needed to improve electrostatic control in the sub-10 nm nodes. Ge-based GAA pFETs [4] and nFETs [5] with inversion-mode (INV) operation have been demonstrated. However, junction formation in Ge INV devices is a critical issue owing to the low dopant solubility, rapid dopant diffusion, and low thermal budget. To solve these issues, junction-less (JL) devices [6] that use a heavy doped channel with the same carrier type as that of the source/drain (S/D) regions have been suggested as alternatives. However, the rapid scaling of transistors requires the development of new and more effective devices that can catch up with modern transistors. In recent years, JL-FETs have been found to be promising as next-generation transistors. The JL-FET is basically a resistor in which the mobile carrier density can be controlled by the gate. In the ON state, a large body current exists, owing to the relatively high doping concentration in the channel region; the surface accumulation current is added to this current The level of doping in the JL-FET needs to be high in order to achieve a suitable current drive, while the device cross-section needs to be small enough such that the device can be turned off. However, in the case of highly doped JL-FETs, the carriers undergo significant impurity scattering, owing to which the drive current is significantly degraded [7]. Furthermore, JL-FETs have the advantages of being simple to fabricate and have high charge mobility and low gate capacitance, in contrast to INV devices [8–12]. Recently, double-gate [13] and body-tied tri-gate [14] Ge JL-FET pMOSFETs were demonstrated on germanium-on-insulator substrates and bulk Si, respectively.

As microelectronic devices continue to shrink and process requirements become ever more stringent, plasma modeling and simulation becomes increasingly more attractive as a tool for design, control, and optimization of plasma reactors [15]. Several techniques are used to simulate the behavior of plasma processes based on the disparity in length and time scales. One of the simulation techniques, computational fluid dynamics (CFD), is widely used to predict the flow fields for engineering design features and to extrapolate experimental limitations. Its modeling has been applied to investigate the flow-mixing phenomena [16], but rare study in etching process. Therefore, this study proposed to characterize in detail thermal flow field of plasma reactors for etching process, and then to deduce numerical parameters that can be beneficial to experiments.

In this work, etching was performed to form suspended epitaxial Ge layers over Si as well as other alloy semiconductors for device integration. The simulated results will be validated by experiments; therefore, initial and boundary conditions as well as parameters in numerical model will be modified to enhance data reliablility. We anticipate that optimal parameters can be obtained by experiments and simulations to improve etching techniques, and fulfill this transistor development by performing higher fabrication process quality as well as lower production cost.

## Methods/Experimental

The starting substrates were SOI wafers with a 70-nm-top silicon layer (p-type, 9–18 Ω cm). The wafers were cleaned using the RCA standard clean 1 (SC-1) and RCA standard clean 2 (SC-2) processes, in order to remove any organics, undesired metals, and particles present. This was followed by rinsing in deionized water and drying in N_2_. The Ge film was deposited in a low-pressure chemical vapor deposition epitaxial reactor (Epsilon 2000, ASM) using 10% GeH_4_ as the precursor. Hydrogen was used as the carrier gas. Before the deposition of the Ge film, an in situ HCl-based pretreatment was performed at 850 °C and 10 Torr to prepare the wafer surface. The substrate temperature was then changed to 400 °C to grow a Ge film on the SOI wafer using 10% GeH_4_. The thicknesses of the deposited Ge films were determined using transmission electron microscopy (TEM, thermal emission Schottky-type, 0.5–200 kV). The crystallinities of the Ge films were examined using X-ray diffraction (XRD) analysis (D8A, Bruker, CuKα radiation, λ = 1.5408 Å, 20–70°). In addition, Ω–2θ scans were performed around the (004) diffraction peak using an X’Pert MRD (PANalytical) system. A two-fold Ge {400} channel-cut crystal collimator was used to select the CuKα1 radiation. During the standard XRD measurements, the sample was fixed in a horizontal position, and the source and detector arm of the diffractometer were moved in the θ–θ mode. Reciprocal space mapping was performed in the medium-resolution mode using the abovementioned crystal collimator; 0.4-mm-wide slits were present on the detector arm in front of the scintillation counter. The thicknesses of the Ge layers were determined via ellipsometry measurements (M2000, J. A. Woollam Co., λ = 193–1690 nm). The fin was formed by anisotropic plasma etching using Cl_2_/HBr gas. After the formation of the ZrO_2_/TiN gate, the S/D were implanted with B (1 × 10^15^ cm^−2^, 15 keV) and activated by rapid thermal annealing at 550 °C for 30 s.

### Highly Selective Dry Etching of Germanium Mechanism

Electron and neutral reactions are isotropic while ion reactions are highly directional and vary with the applied bias. By adjusting the bias power, the ions can be accelerated along the desired direction to aid the etching reaction. It is generally accepted that Br does not react spontaneously with Si and that energetic ions are needed for the reaction to occur. Ion-assisted Br and Cl atoms can react with Ge or Si atoms spontaneously under activation to form the volatile products GeBr_4_, GeCl_4_, SiBr_4_, and SiCl_4_, which are desorbed from the substrate surface and can be pumped away. This ion-assisted chemical reaction of Si with Br has been shown to be highly anisotropic. Higher vertical etch rates were obtained using HBr for bias power variations, which confirms that addition of HBr in Cl_2_ can enhance the etch rate [17]. Since Cl- and Br-based plasma etch Ge and Si by an ion-assisted mechanism, the energy of ions such as Br^+^, Br^2+^, HBr^+^, Cl^+^, and Cl2^+^ can be controlled by biasing the substrate holder. There is no pronounced change of lateral etch rate by varying the bias power for both etching at top surface and necking area. Undercuts were found in the fin structures etched using HBr mixtures, in the necking area, since lateral etching was enhanced by the defects along the Ge/Si interface. And also the mask potentially influences the shape evolution due to the sidewall striking from ion flux. This phenomenon is amplified as the profile becomes deeper and as the lateral ion velocity component increases. Because of the high angular dependence of the HBr-plasma etching process [18], thus, it can be concluded that the crystal structure is relatively weaker at the Ge/Si interface because of the partially bound atoms from the misfit dislocations and the relatively weak Ge-Ge and Ge-Si bonds. By adjusting the ratio of HBr/Cl_2_ and bias power, different types of fin-like structures can be obtained during Ge device fabrication. The etching properties of Ge and Si are very similar. Gases that etch Si usually etch Ge at a greater rate. The strengths of Ge and Si bonds have been shown to be Ge-Ge = 263.6 ± 7.1 kJ mol^−1^ and Si-Si = 325 ± 7 kJ mol^−1^ [2]. Different bias powers were applied to adjust the vertical etch rate by directional acceleration of the ions. Images were recorded after the gate stack process. The vertical etch rate was calculated from the film height relative to the oxide substrate. Lateral etch rates were determined from two parts of the fin: the lateral etching at the top surface of the fin, and the etching in the necking area close to the Ge/Si interface. In summary, the bias power was experimentally found to be the most critical parameter in the etching process and therefore, in affecting the device characteristics accordingly.

In this study, all the etching processes were performed in a TCP 9600 reactor from Lam Research. This is a transformer-coupled plasma reactor that allows for separate control of the coil (top electrode) power and the substrate (lower electrode) bias. Helium backside cooling was incorporated to allow the temperature of the substrate to be controlled more effectively. The samples were mounted on a 6-in. Si carrier wafer with vacuum grease before being introduced into the etching chamber. HBr/Cl_2_ chemistry was exploited for the anisotropic etching process. The process pressure was controlled at 10 mTorr. The detailed experimental set-up and conditions are illustrated in the study of Hsu et al. [4].

### Physical Model

**Fig. 1:**
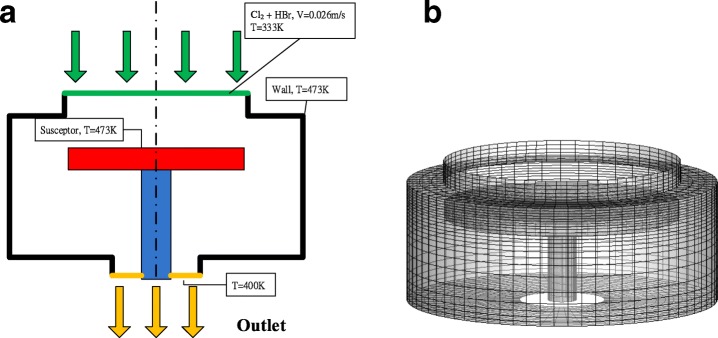
Physical model for numerical simulation of plasma reactor. **a** The sketch of the reactor chamber with prescribed boundary conditions. **b** Mesh system for numerical simulations

CFD is used to access the internal information inside the reactor and the flow is considered to be laminar with the slip boundary condition due to higher Knudsen number in the chamber (Fig. 1). In the present simulation, plasma thermal flow is considered as mixture in the momentum equations; particle transport is considered in the species governing equations and chemical reactions are involved on the susceptor wall. One model case for simulation is illustrated in Fig. 3. The inlet gas is a mixture of chlorine (Cl_2_) and hydrogen bromide (HBR). The mixture velocity at the inlet is 0.026 m/s with temperature kept at 333 K. The susceptor and the chamber wall are heated to operating temperature 473 K. These plasma flow drived by electric potential bias and have chemical reactions on the susceptor surface in a uniform, repeatable manner, and finally to be discharged from the reactor. After build the solid modeling by the CAD software, the mesh system was constructed in structured grids with 50, 000 nodes as shown in Fig. 3. The transient conservation equation for transport of a scalar quantity ϕ is demonstrated by the following equation written in integral form for an arbitrary control volume V as follows [19]:

1* $$ \frac{d}{dt}\underset{V}{\oint}\rho \varphi\;dV+\oint \kern0.27em \rho \varphi \overset{\rightharpoonup }{v}\cdot \mathrm{d}\overrightarrow{\mathrm{A}}=\oint \Gamma \mathrm{\nabla}\varphi \cdot \mathrm{d}\overrightarrow{\mathrm{A}}+{\int}_{\mathrm{V}}{\mathrm{S}}_{\varphi}\mathrm{dV} $$

where ρ indicates density; $\overset{\rightharpoonup }{v}$ is velocity vector; $\overset{\rightharpoonup }{A}$ is surface area vector; V is volume; Γ is the diffusion coefficient for ϕ and S_ϕ_ is source term of ϕ per unit volume. The symbol ϕ can be replaced by 1 for the continuity equation, *u*, *v*, and *w* are the momentum equations in the X, Y, and Z directions, and Ci for the species transport equations in the reactor chamber respectively. Eq. (1*) can be expressed in generalized coordinates using the finite volume method approach for simulation. After combining boundary conditions, Eq. (1*) can be linearized and expressed in the computational domain as a set of algebraic equations, which can be solved by the SIMPLE algorithm using the CFD technique [19]. Noted that most semi-conductor fabrication devices operate far below atmospheric pressure. At such low pressures, the fluid flow is in the slip regime and the normally used no-slip boundary conditions for velocity and temperature are no longer valid. The present plasma flow at very low pressure is in the slip regime, which is between free molecular flow and the continuum regime [20]. Therefore, slip boundary conditions for velocity and temperature for modeling fluid flow are imposed in our numerical simulations.

The semiconductor materials Si(s) and Ge(s) are etched away on the heated susceptor surface governed by the following surface reactions:

2*.1 $$ {\mathrm{Cl}}_2+\mathrm{Si}={\mathrm{SiCl}}_4 $$

2*.2 $$ 4\mathrm{HBr}+\mathrm{Si}={\mathrm{SiBr}}_4+2{\mathrm{H}}_2 $$

2*.3 $$ 2{\mathrm{Cl}}_2+\mathrm{Ge}={\mathrm{GeCl}}_4 $$

2*.4 $$ 4\mathrm{HBr}+\mathrm{Ge}={\mathrm{GeBr}}_4+2{\mathrm{H}}_2 $$

The chemical reactions are similar for Si etching in Eq. (2*.1) and Eq. (2*.2), or Ge etching in Eq. (2*.3) and Eq. (2*.4). Hence, Si etching process by the mixture of chlorine and hydrogen bromide are demonstrated in the following simulations.

## Results and Discussion

### Material Characterization

**Fig. 2:**
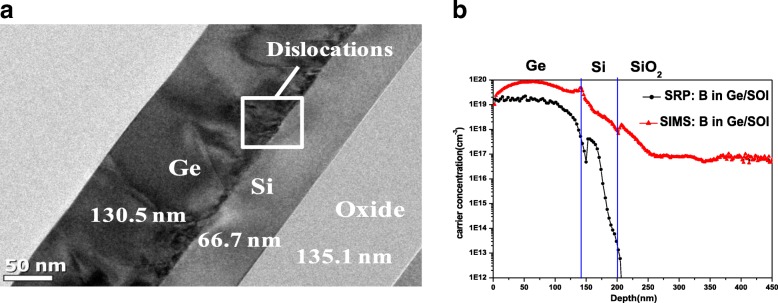
**a** The TEM image of the p-type Ge on SOI substrate. **b** The SIMS and SRP profiles of the in situ boron doped epi-Ge layer on SOI. The hole concentration is low in the bottom defective Ge near Ge/Si interface

**Fig. 3:**
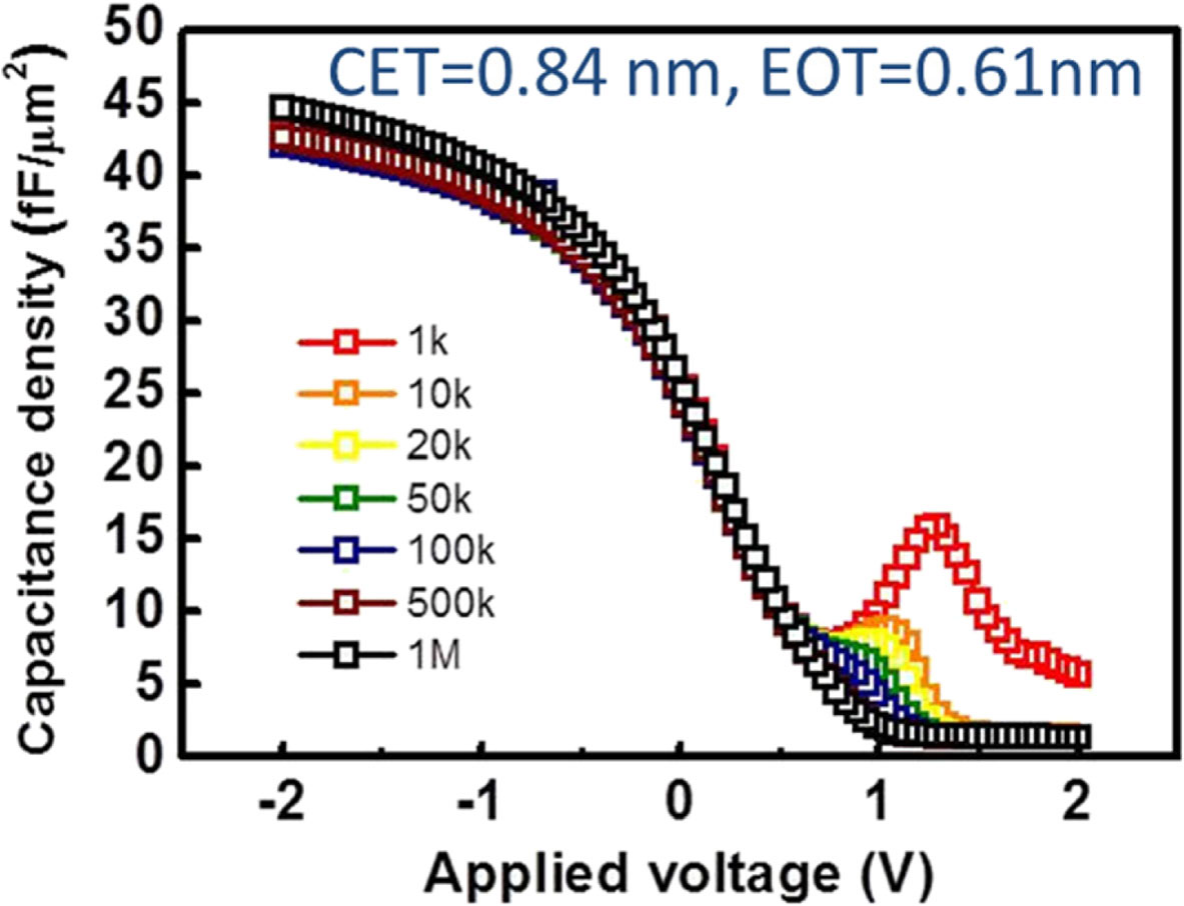
C-V characteristics of the TiN/ZrO_2_/Ge MISCAPs with EOT ~ 0.6 nm

**Fig. 4:**
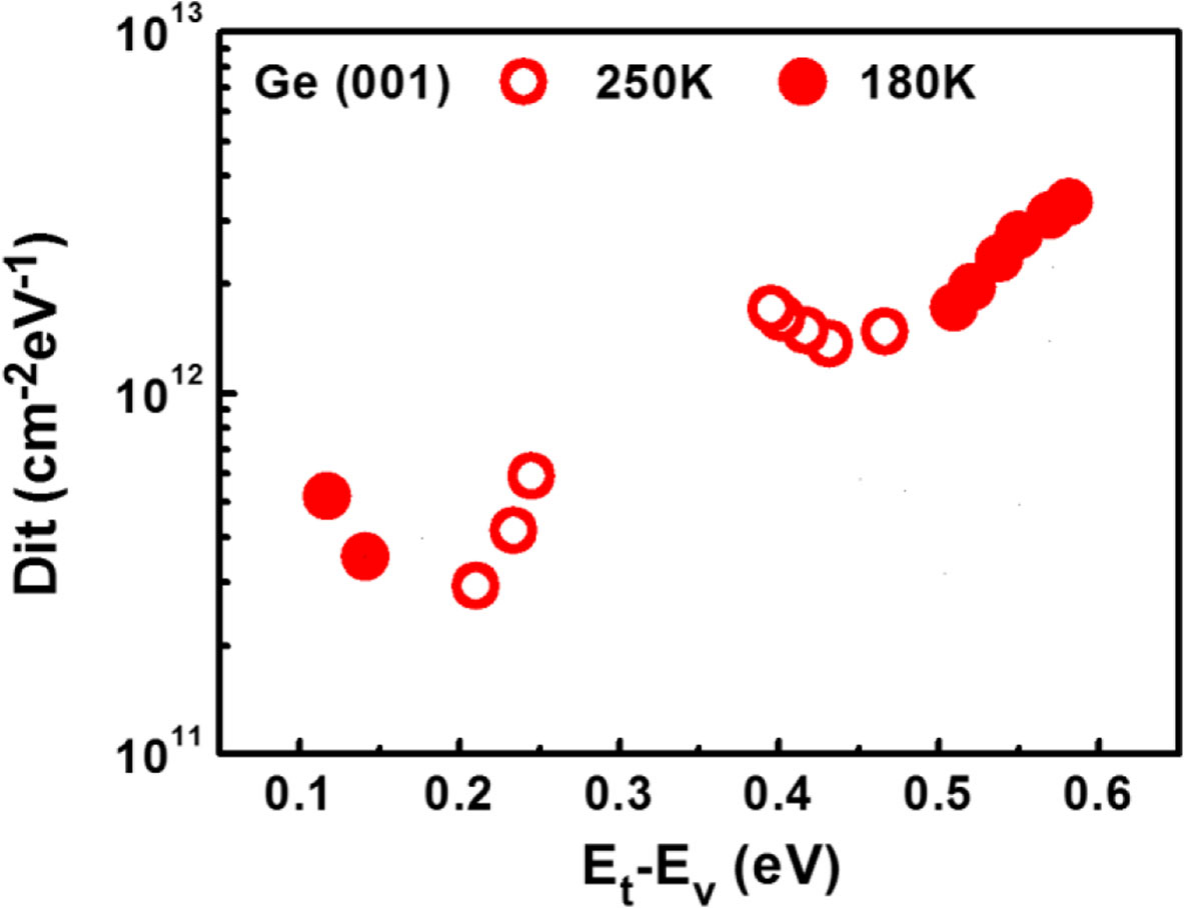
Dit measured by low temperature conductance method

Cross-section TEM images of the Ge layer formed on the SOI substrate are shown at Fig. 2a. As can be seen, misfit dislocations are present at the Ge/Si interface; these, in turn, result in threading dislocations across the epitaxial Ge film. These threading dislocations are thought to accommodate the thermal mismatch between Ge and Si. Most of the threading dislocations terminated within 80 nm from the interface; however, many also propagated to the film surface. The epitaxial Ge film on the SOI substrate was implanted with boron and subsequently activated, in order to examine the dopant distribution and the activation level. For the top 130 nm part of the Ge layer, the boron activation rate was ∼ 85%, as shown in the PCOR-SIMS and spreading resistance profiling (SRP) profiles. (see Fig. 2b). The hole concentration dropped significantly in the case of the bottom of the Ge layer near the Ge/Si interface, owing to the presence of defects and because of the measurement limits of SRP. In the case of the fabricated devices, the defective Ge near the Ge/Si interface was removed by selective etching; thus, the PCOR-SIMS and SRP measurements performed in the channel should be accurate. The maximum activation level of **~** 3 × 10^19^ cm^−3^ is completely consistent with the conventional activation limit. Note that owing to the low-temperature activation (550 °C) performed during boron implantation, the S/D near the parasitic Si channel were highly resistive (Fig. 2); this prevented parasitic Si conduction. Figure 3 shows the capacitance-voltage (CV) characteristics of the TiN/ZrO_2_/Ge metal-insulator-semiconductor capacitors (MISCAPs). To prevent the formation of an unstable GeO_x_ layer during the high-k dielectric deposition and post-deposition annealing process, the nitride-based material Ge_3_N_4_ and not GeO_2_ was inserted as the interface layer on the Ge (001) surface through a NH_3_/H_2_ remote plasma treatment. The ZrO_2_ layer was deposited at 250 °C for 20 cycles by atomic layer deposition. The measured CV curves do not indicate frequency dispersion or stretch-out from 1 KHz to 1 MHz and are consistent. The loss, which creates trap levels near the ZrO_2_/Ge interface, shifts the thermal activation energy for minority carrier generation from that corresponding to the p-Ge bandgap energy to the midgap energy. The equivalent oxide thickness (EOT) is 0.6 nm and the *D*_it_ (Interface-trap density) value is ~ 3 × 10^12^ cm^2^ eV^−1^ near the midgap, as measured by the low-temperature conductance method (see inset of Fig. 4).

### Simulation for the Parameters of Operating the Reactor

**Fig. 5:**
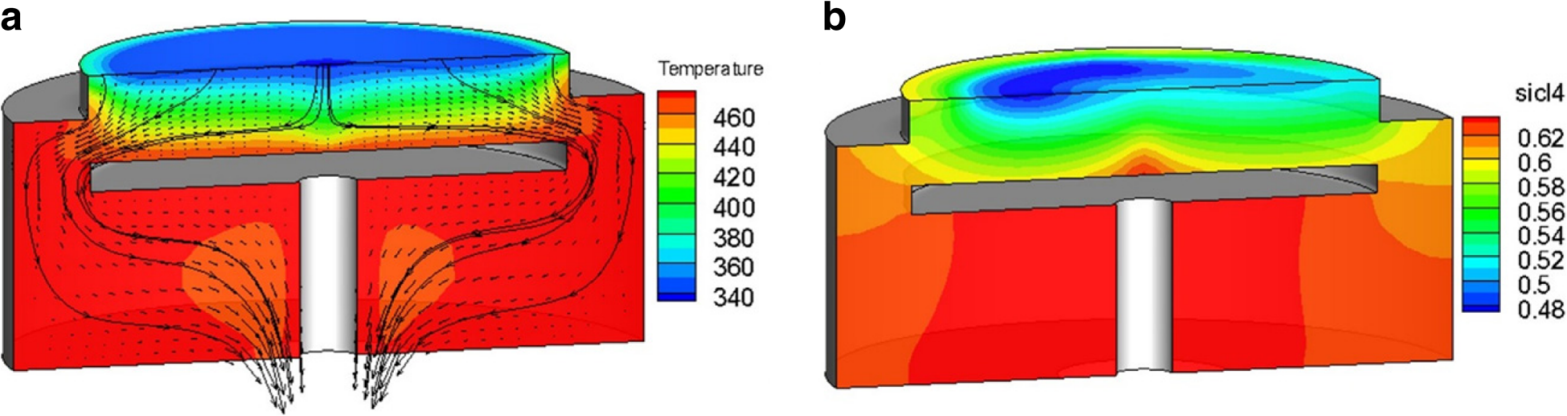
Contours of plasma parameters inside the reactor. **a** Temperature distribution and particle path lines r. **b** Mass fraction contours of product SiCl_4_

**Fig. 6:**
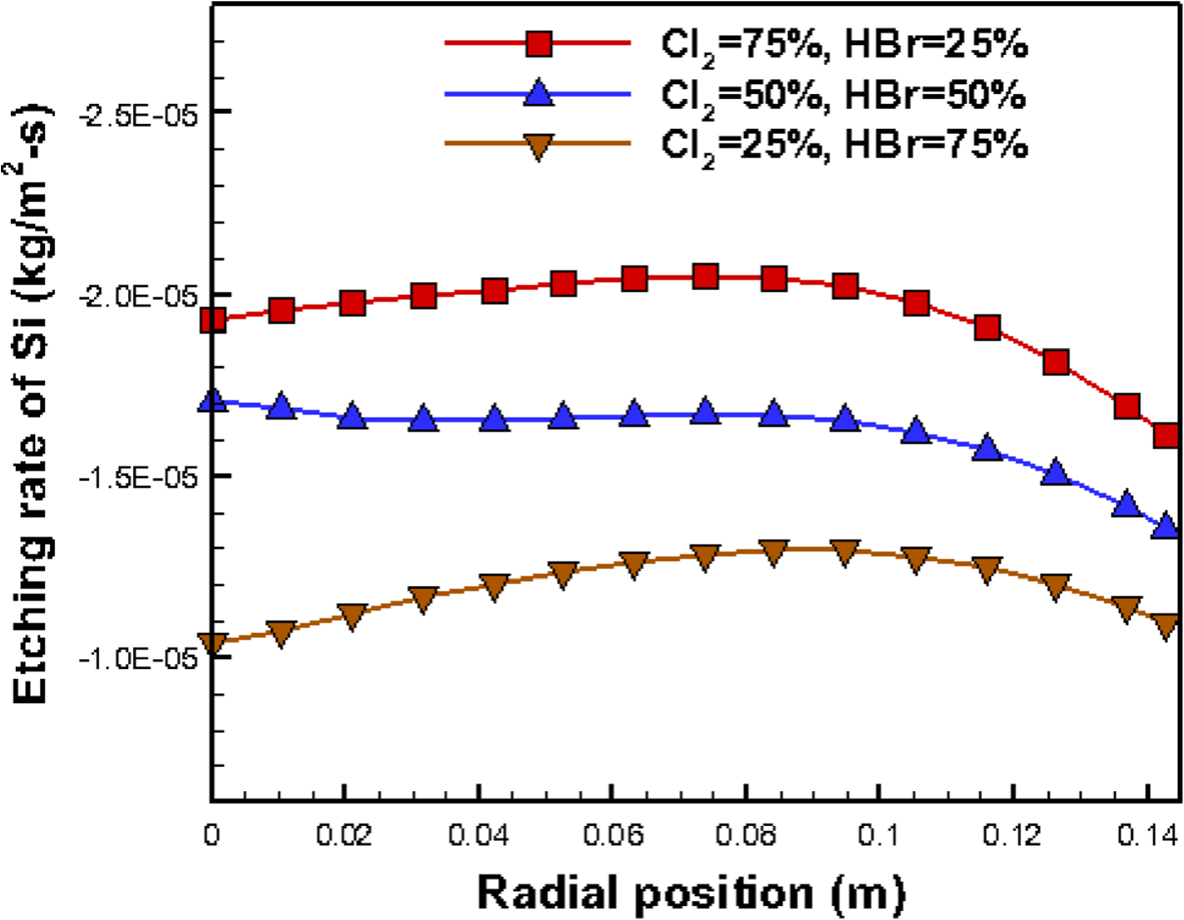
Etching rates of Si on the susceptor from mixtures of inlet gases with various fractions

A typical model of steady laminar plasma flows was simulated on high speed personal computers. The inlet gas is a mixture of Cl_2_, which has a mass fraction of 0.75, and HBr, which has a mass fraction of 0.25. Figure 5a illustrates contours of the low temperature inflow, operating higher temperature inside and the particle path lines in the reactor chamber. Figure 5b shows the mass fraction contours of product SiCl4, which has low concentration above the susceptor and has high concentration below the susceptor to the exit. Besides, higher mass fraction of Cl_2_ gets good performance in etching, and this know-how has been validated by present simulations as shown in Fig. 6. The horizontal axis represents along one radial position on the susceptor and the vertical axis indicates etching rate (kg/m^2^ s) of Si. Figure 6 shows that the better etching process is archived by the inlet mixture of 75% Cl_2_ and 25% HBr, and this mixture was adopted to conduct experiments in this study.

**Fig. 7:**
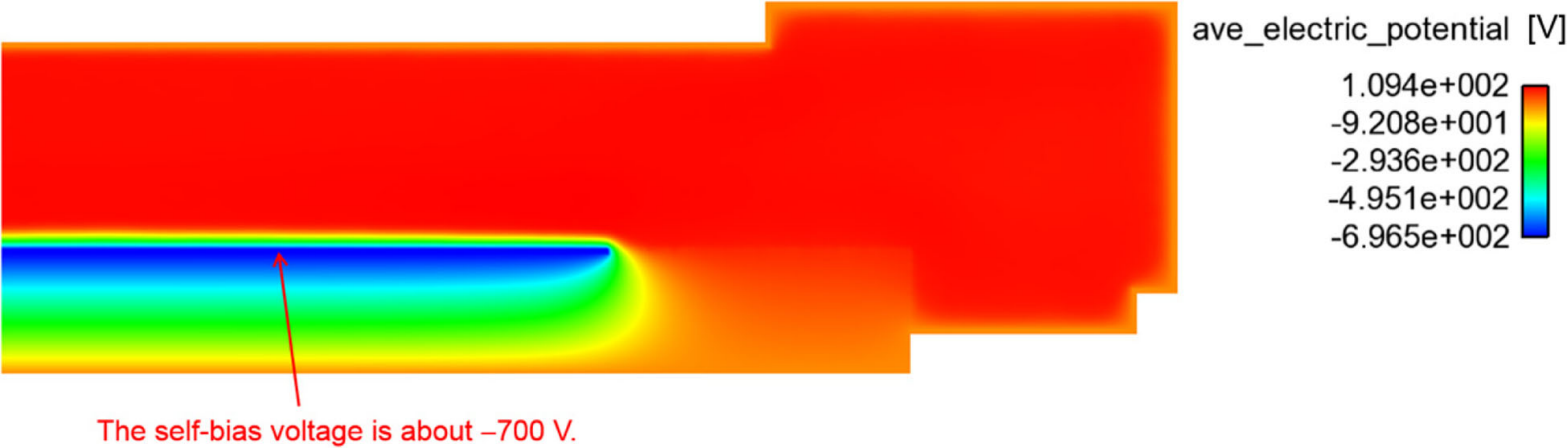
Simulation of electric potential distribution for plasma etching in the chamber

Another case to show the feasibility of computer aided experiment is electric potential distribution in the chamber for plasma etching. Based on the underlying mechanism of this etching process, a 2D simulation model was developed for the distribution of the plasma power density as shown in Fig. 7 and was used to fit the measurement data, in order to confirm the accuracy of the model and assist the experiment.

As proposed by Sugai [21], the self-bias voltage can be related to capacitances of sheath on RF electrode (C_K_) and earth electrode (C_A_), and expressed as follows:

3* $$ {V}_{\mathrm{DC}}=\frac{C_{\mathrm{K}}-{C}_{\mathrm{A}}}{C_{\mathrm{K}}+{C}_{\mathrm{A}}}\ {V}_{\mathrm{RF}} $$

According to present simulations as shown in Fig. 7, the self-bias voltage is about − 700 V, which is close to the theoretical value, − 650 V, as calculated from Eq. (3*). Therefore, it is suggested that optimization be performed using simulations in order to not only adjust the process parameters used but also to modify the hardware employed. This will help ensure uniformity over the entire run and lead to high-quality, low-cost processes that are optimized.

### Device Fabrication and Characterization

**Fig. 8:**
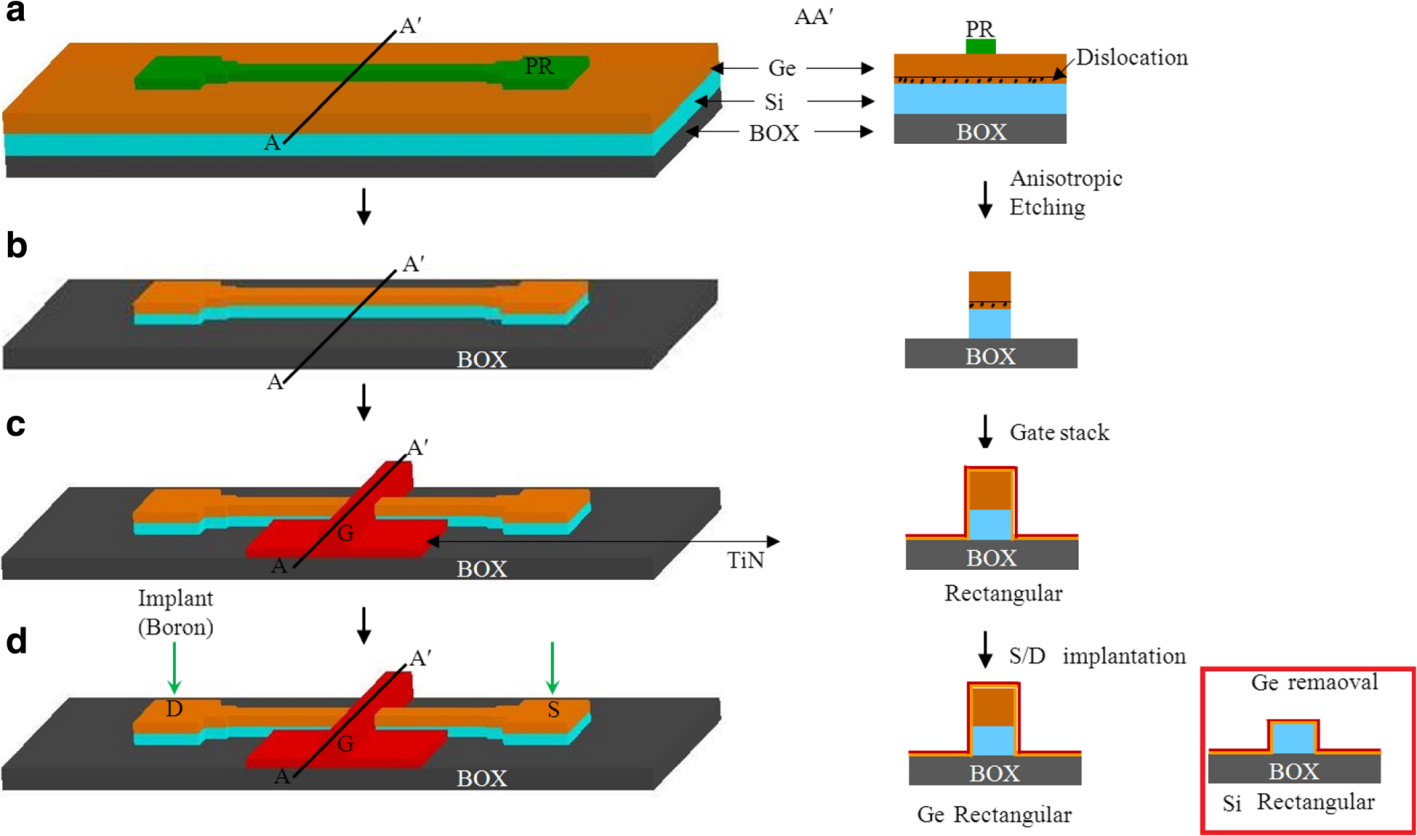
Schematic of the device fabrication. **a** Fin patterning. The starting materials is Ge (130 nm) on BOX. **b** Anisotropic etching and photoresist striping. **c** Gate formation by atomic layer deposition of ZrO_2_ and TiN deposition. **d** Self-aligned boron implantation on S/D for good contact. Note: the left side is the 3D schematics and the right side is the corresponding cross sectional view

**Fig. 9:**
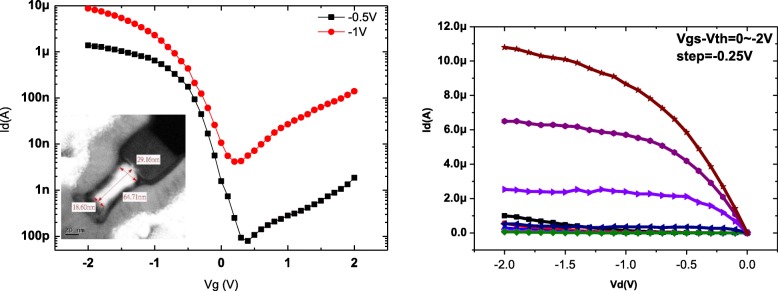
*I*_d_–*V*g and *I*_d_–*V*_d_ curve for the Ge FinFET

**Fig. 10:**
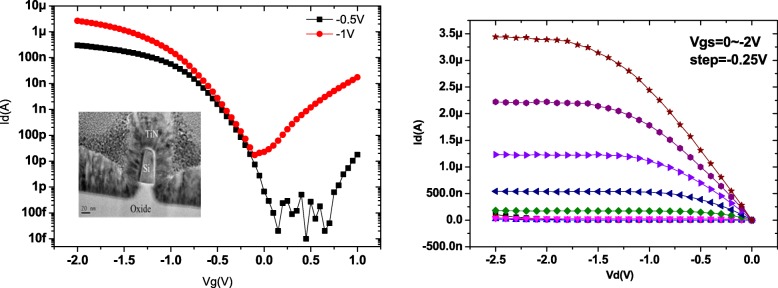
*I*_d_–*V*g and *I*_d_–*V*_d_ for the Si FinFET after the top Ge is carelessly etched away. Although *I*_on_/*I*_off_ can reach 10^8^, its on current value is very low

**Fig. 11:**
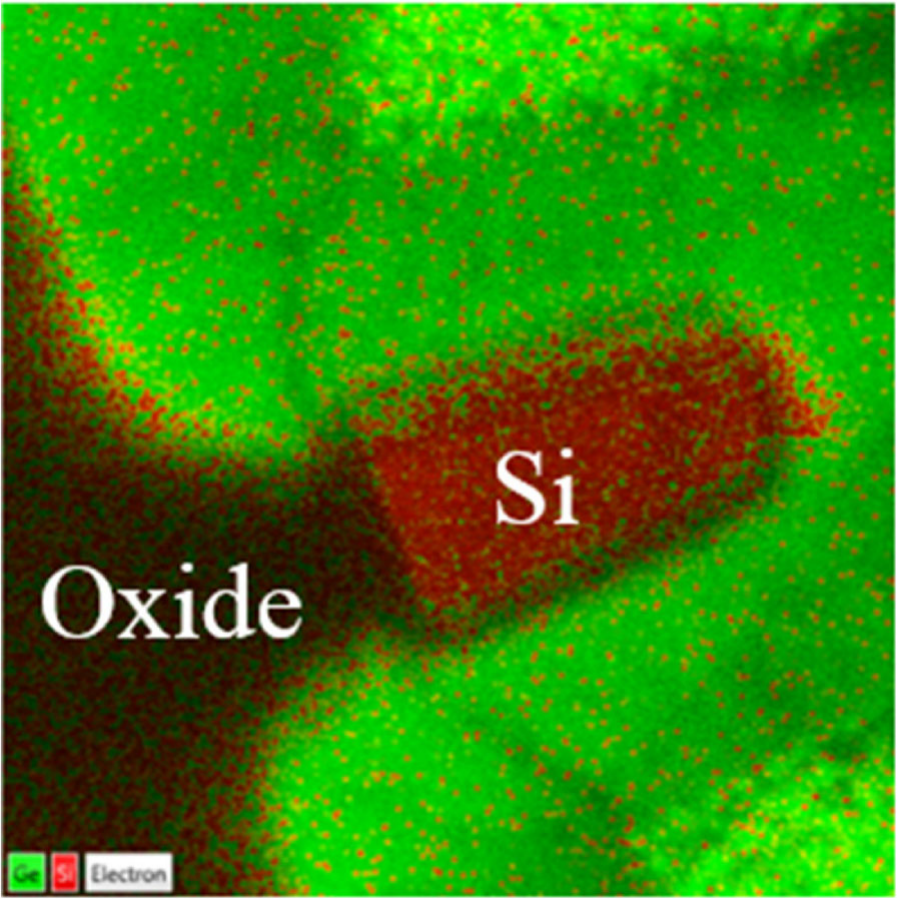
TEM mapping for the channel structure that undergoes an over-etching process

The epitaxial Ge layer was patterned into fins with the desired feature sizes using e-beam lithography. The fin was formed by anisotropic etching with Cl_2_/HBr-based plasma to etch away the high-defective Ge near Ge/Si interface. The floating Ge fin on SOI was formed with the higher etching selectivity of Ge than Si and the enhanced etching rate of the defective region [4]. A SiO_2_ capping layer was then deposited and became the spacer after gate patterning. After defining the active region, the gate stacks of ZrO_2_ layer were formed by ALD, respectively. The channel cross sections of fabricated devices are shown in Fig. 8. The fin width (*W*_fin_) is used for the channel concentration of 8 × 10^19^ cm^− 3^ which is extracted using the van der Pauw method on a blanket Ge epi layer on SOI. Note that the channel controllability decreases with increasing channel concentration and increasing *W*_fin_ [12, 13]. The large channel concentration requires the small *W*_fin_ to maintain the low SS. Finally, the gate electrodes were defined and deposited. Figure 9 shows the output and transfer characteristics of a triangular Ge FinFET with a fin width (*W*_fin_) of 18 nm and gate length (*L*_g_) of 90 nm. The *I*_on_/*I*_off_ ratio of the Ge JL-FET is as high as 10^5^ and the subthreshold swing (SS) is ~ 100 mV dec^− 1^. The transfer characteristics of the Si JL-FET are shown in Fig. 10. The *I*_on_/*I*_off_ ratio of the Si JL-FET is high as ~ 10^8^, its SS is 90 mV dec^− 1^, its *L*_g_ is 80 nm, and its *W*_fin_ is 20 nm. Figure 8 also shows that the span of the gate voltage, Δ*V*_g_, is approximately 0.5 V and close to the bandgap of Ge (*E*_g_/*q* = 0.66 V). This confirms that the *I*_d_–*V*_g_ curves shown in Fig. 8 are for a Ge FinFET. However, the span of the gate voltage, Δ*V*_g_, shown in Fig. 6 is approximately 1.8 V and close to the bandgap of Si (*E*_g_/*q* = 1.1 V) but not that of Ge (*E*_g_/*q* = 0.66 V). Thus, the *I*_d_–*V*_g_ curves shown in Fig. 9 are for a Si JL-FET and not a Ge JL-FET. This observation is based on semiconductor device physics and is supported by the experimentally determined *I*_d_–*V*_g_ curves of the Ge and Si FinFETs as well as the cross-sectional TEM/EDS Si maps. Two things are evident from the insets in Fig. 11. The insets show the output and transfer characteristics of the unexpected Si JL-FET after Ge overetching; the *I*_on_/*I*_off_ ratio of this device is as high as 10^8^. However, its ON current is only 17 μA μm^− 1^ at − 1 V. The high *I*_on_/*I*_off_ ratio is attributable to the Si layer and not the Ge layer. When only the Si layer is left, the device is actually a Si JL-FET rather than a Ge JL-FET.

## Conclusions

We confirm the relationship between simulation model and measurement data. Therefore, it is suggested that optimization be performed using simulations in order to not only adjust the process parameters used but also to modify the hardware employed. By the help of numerical simulations to determine the operating parameters for the rector, we showed that the parameters for the etching process for forming Ge/Si channels can be optimized through experiments, in order to improve the etching process and aid the development of transistors by improving the fabrication quality and lowering the production cost. The experimental results indicated that the dry etching technique developed for Ge FinFETs is also extremely useful for the fabrication of tall-fin CMOS devices.
